# Empathy, Affect and Personality as Predictors of Engagement in Nursing Professionals

**DOI:** 10.3390/ijerph18084110

**Published:** 2021-04-13

**Authors:** África Martos Martínez, María del Carmen Pérez-Fuentes, María del Mar Molero Jurado, María del Mar Simón Márquez, Ana Belén Barragán Martín, José Jesús Gázquez Linares

**Affiliations:** 1 Department of Psychology, Faculty of Psychology, University of Almería, 04120 Almería, Spain; amm521@ual.es (Á.M.M.); mpf421@ual.es (M.d.C.P.-F.); mmj130@ual.es (M.d.M.M.J.); msm112@ual.es (M.d.M.S.M.); 2 Department of Psychology, Universidad Autónoma de Chile, Providencia 7500000, Chile; jlinares@ual.es

**Keywords:** empathy, affect, personality, engagement, nursing

## Abstract

Background: It seems that personality traits affect engagement and the quality of professional life, which is mediated by the emotional and affective states of nursing personnel. Therefore, the objectives of this study were to analyze the relationships between the components of empathy, affect, personality, and engagement, find personality profiles, identify the variables with the most explanatory value, and analyze the mediating role of the variables susceptible to intervention in the relationship between personality and the components of engagement. Methods: A sample of 1268 nurses completed the Utrecht Work Engagement Scale, 10-item Big Five Inventory, Basic Empathy Scale, and Positive and Negative Affect Schedule. Results: Empathy, affect, and personality influence engagement factors in nurses. The vigor and absorption factors of engagement showed a positive relationship with empathy, positive affect, and all of the Big Five personality factors except neuroticism with which the relationship was negative. Personality affected the vigor, dedication, and absorption factors of engagement, and cognitive empathy mediated this relationship. Conclusions: This study shows the need to continue investigating the factors that affect and mediate in engagement of nursing professionals.

## 1. Introduction

In recent years, interest in the study of wellbeing and job commitment, or engagement, of healthcare workers has increased due to its influence on their intention of quitting the job and hospital service quality [1,2,3]. Engagement is closely related to job satisfaction and performance [4,5]. Thus, some studies note that the development of intervention directed at improving engagement generates greater efficacy in providing services than those intended to reduce burnout in nursing [6].

Engagement refers to a positive, fulfilling work-related state of mind, which is characterized by vigor (high levels of energy and mental resilience while working), dedication (experiencing a sense of meaning, enthusiasm, and challenge in professional duties), and absorption (being fully concentrated on one’s work) [7,8]. Showing a high level of engagement does not mean ignoring negative aspects of the work environment. However, nursing professionals who maintain high levels of engagement see positive aspects, such as enjoying their work, the meaning of being a nurse, and the rewards perceived, enabling them to overcome the burnout process [9].

Even though engagement is closely related to the specific characteristics of the task performed, if the worker has the personal resources, they provide sufficient motivation and energy to achieve professional engagement [10]. Thus, various individual factors, such as emotional intelligence [11], creativity [12], level of involvement in the work [13], agreeableness, conscientiousness, emotional stability [14], self-efficacy [15,16], and perception of a climate of psychological safety [17] have been associated with the level of engagement of professionals.

### 1.1. Empathy, Affect, and Personality in Nursing Professionals and Their Relationship to Engagement

Some people show individual strengths that facilitate coping with the stress and professional challenges typical of jobs such as nursing [18]. Thus, according to the Big Five model [19], nursing professionals with a resilient personality are emotionally stable, extroverted, and communicative [20]. Nurses who identified with this type of personality report less job stress [21]. Following this model, several personality profiles have been found in nursing. These profiles show a different association with burnout and job engagement. Specifically, nursing professionals with a well-defined profile based on high scores in neuroticism and low in the rest of the personality traits are more vulnerable to burnout, whereas nurses with the opposite profile (high scores in extraversion, agreeableness, openness to experience and conscientiousness, and low in neuroticism) show strong engagement [22]. Along this line, Van Mol et al. [14] mentioned the existence of a negative relationship between the presence of neurotic traits and engagement in nursing professionals.

Other individual variables that also seem to have a strong association with engagement in nursing are empathy and affect. Empathy is the ability to grasp the framework of reference of another, and it is a key characteristic in nursing practice, involving self-awareness and the use of emotion in interpersonal understanding [23]. Therefore, this variable is considered a basic element in the wellbeing of nursing professionals [24,25], in therapeutic relationships, and in the quality of care given to the patient [26]. It is also one of the ten attributes considered basic in the constitution and development of the nursing profile [27]. Thus, in recent years, there has been an increase in interventions promoting the acquisition of these attributes by nurses in training [28], especially as empathy training programs tend to be effective [29]. Nevertheless, as demonstrated by Macysiak, Dabrowska, and Marcysiak [30], empathy is comprised of one cognitive dimension and another affective. While the first improves job satisfaction and work engagement and reduces the intention of quitting the organization voluntarily, the affective dimension does not generate a positive impact on the nurse’s wellbeing. Thus, empathy, especially the cognitive attributes (understanding, communication, suitable response), modulates the influence of stress and becomes essential to the quality of the therapeutic relationship and the experience of nursing professionals [31]. Furthermore, it protects these workers from developing burnout [32,33].

Another emotional variable of great importance in nursing is affective experience. More and more emphasis is being placed on enriching the affective domain in their professional training [34,35]. Affect is related to patient perception of service quality and general wellbeing of healthcare workers [36]. According to Watson, Clark, and Tellegen [37], emotional experience is divided into positive and negative affect [38]. An individual with high positive affect has a general tendency to experience positive moods more intensely and more often, while an individual with high levels of negative affect experiences negative moods to a greater extent. Thus, the presence of high levels of affect leads to developing personality virtues, such as humanity and calmness [39], and it promotes mental health [40]. Whereas, when nursing professionals perceive that the workload is excessive and they cannot give quality care to the physical and emotional demands of the patients, feelings such as sadness or negativity appear. In turn, this perception of inability to cope with the demands of the job negatively affects workers’ vigor [41].

A model linking emotional affect and engagement [42] has recently been proposed. This model shows that the level of engagement is related to the greater or lesser presence of certain affective states. Thus, having strong engagement is linked to intensely experiencing positive affective states. As the level of engagement falls, the intensity of emotions is also reduced, and some negative emotional states appear. Finally, the absence of occupational engagement is related to emotional disconnection. However, this model has been tested only in students in the area of technology.

### 1.2. Positive Affect and Cognitive Empathy as Personality Mediators on Engagement

In addition to clinical competence, in the nursing profession, personal emotion management is essential to meet work demands [43]. Similar to other collectives [44], nursing professionals employ balanced strategies for emotional coping based on high levels of cognitive empathy and low emotional empathy. This enables them to understand patients and family members, adopting a protective reaction for their own emotional health, which permits them to distance themselves and cope with the emotional demands of the work environment [14]. The use of this type of strategy is associated positively with engagement absorption levels, while the presence of high levels of personal distress in nurses when observing the anguish of others, which is linked to the more emotional part of empathy [45], is related to less dedication [46]. In this line, the study by Cao and Cheng [47] showed that the engagement of nurses in hemodialysis was strongly influenced by their level of cognitive empathy.

In this regard, the Big Five personality model seems to be a significant predictor of empathy shown by nursing professionals [48], as well as affect [49]. Specifically, the agreeableness and neuroticism personality traits influence burnout and stress levels. However, this relationship is not direct, but it is mediated by positive affect in the case of burnout and by negative affect when it is stress [50]. In this line, positive affect has been shown to be a partial mediator of engagement and job resilience [51].

At the present time, due to socio-occupational changes, the levels of work engagement in nursing must be increased [52]. According to Lu, Zhao, and While [53], in an explanation of satisfaction and work engagement in nursing, it is indispensable to consider the mediators affecting them. Thus, it seems that personality traits affect engagement and quality of professional life, which is mediated by emotional and affective states of nursing personnel [50]. Based on the above, the objectives of this study were the following: (1) analyze the relationships between the components of empathy (cognitive/affective), affect (positive/negative), personality, and engagement in a sample of nursing professionals, (2) find personality profiles and determine whether there are statistically significant differences in empathy, affect, and engagement, (3) identify the variables with the most explanatory value in the dimensions of engagement, and (4) analyze the mediating role of the variables susceptible to intervention (affect and empathy) in the relationship between personality and the components of engagement.

## 2. Materials and Methods

### 2.1. Participants

The original sample was made up of 1383 nursing professionals (Andalusia, Spain). First, with a view to using engagement as one of the main variables in the study, all those unemployed at the time of data collection (−68 subjects) were excluded. Then, by analyzing the answers to control questions (CQ), those cases in which incongruent or random answers were detected were also discarded. Specifically, five subjects failed CQ_2_, 32 failed CQ_3_, and 10 failed CQ_5_. After filtering, the sample was finally comprised of 1268 nurses. The mean age of the participants was 32.02 (*SD* = 6.91), in a range of 22 to 63 years. By sex, the distribution of the sample was 14.7% (*n* = 187) men and 85.3% (*n* = 1081) women, with a mean age of 32.79 years (*SD* = 6.27) and 32.24 years (*SD* = 6.68), respectively.

### 2.2. Instruments

The Utrecht Work Engagement Scale (UWES) [54] was used to evaluate work engagement. This is a 17-item self-report scale, with answers rated on a seven-point Likert-type scale. It provides information on three dimensions of engagement: Vigor (“At my work, I feel bursting with energy”), Dedication (“I find the work that I do full of meaning and purpose”), and Absorption (“Time flies when I’m working”). Adequate reliability and validity have been found for this test [55]. In this case, with a sample of nursing professionals, the internal reliability found was adequate for each of the dimensions: α = 0.89 in Vigor, α = 0.91 in Dedication, and α = 0.85 in Absorption.

The 10-item Big Five Inventory was used to evaluate personality dimensions (BFI-10) [56]. This is a brief version of the BFI-44 [57,58], which was developed to provide a personality inventory in research environments with limited time, and it is adequate for studies such as this one, in which data are collected on a wide diversity of variables. It enables approximate information to be found and evaluated on the Big Five personality factors: extraversion (“I see myself as someone who has few artistic interests”), responsibility (“I see myself as someone who does a thorough job”), agreeableness (“I see myself as someone who is generally trusting”), neuroticism (“I see myself as someone who gets nervous easily”), and openness to experience (“I see myself as someone who has an active imagination”). Previous studies have shown that the BFI-10 has psychometric properties comparable in size and structure to the full BFI scale. There are findings backing the factorial, construct, and criterion validity of the BFI-10 [56,59,60].

Empathy was measured using the Basic Empathy Scale (BES) [61], as adapted by Oliva et al. [62]. It consists of nine items distributed in two scales corresponding to affective (“After I am with a friend who is sad for some reason, I usually feel sad”) and cognitive empathy (“When someone is depressed, I usually understand how they feel”). These elements are answered on a five-point Likert-type scale. The scores are found by simply adding up the items, where higher scores are interpreted as more intensity in empathic behavior. The internal consistency reported by Oliva et al. [60] for both scales was 0.73 and 0.63, respectively. In this study, the internal consistency found with the Cronbach’s alpha was 0.84 for both scales.

Finally, the Spanish adaptation (SPANAS) [63] of the Positive and Negative Affect Schedule (PANAS Scale) [37] was used to evaluate positive and negative affect. This test consists of 20 items organized in two groups, 10 on positive affect (e.g., enthusiastic, active, and alert) and 10 on negative affect (e.g., upset, guilty, and afraid). The answers are rated on a Likert-type scale from 1 to 5 (where 1 = very little or not at all, and 5 = very much). The original version by Watson et al. [37] showed adequate psychometric properties, with 0.86 for positive affect and 0.85 for negative. The Spanish adaptation was somewhat lower with a reliability of 0.78 for positive affect and 0.75 for negative affect [63].

### 2.3. Procedure

Prior to collecting data, we assured the participants that the treatment of data in the study would comply with applicable standards of data security, confidentiality, and ethics. The study was approved by the Bioethics Committee. Data were collected using a CAWI (Computer-Aided Web Interviewing) survey, which included, in addition to the validated questionnaires, a series of questions for sociodemographic data. The survey was distributed over the social networks (snowball sampling). Participation was voluntary, and before answering the questionnaire, participants were given information on the study and its purpose on the first page, where they also had to check a box indicating their informed consent before starting to complete the survey. A series of control questions were included to monitor for random or incongruent responses, which were removed from the study.

### 2.4. Data Analysis

This study employed a cross-sectional descriptive and correlational design. First, to identify the relationships between the individual variables (empathy, affect and personality) and engagement, the Pearson’s correlation coefficient was calculated, in addition to the corresponding descriptive statistics. A two-step cluster analysis was also performed to determine the personality profiles. Once the groups or clusters had been identified, a comparison of means was done to determine the existence of significant differences between the groups with respect to the engagement components, using the Student’s *t*-test for independent samples, and the Cohen’s d [64] to find the effect size of those differences. In addition, to find out how the predictor variables (Empathy: affective and cognitive, Affect: positive and negative) were related to the criterion variable, a stepwise multiple linear regression analysis was carried out. Finally, a multiple mediation model was computed for each of the dimensions of engagement, taking the personality profile as the predictor variable. The SPSS macro [65] was used to compute the mediation models. Bootstrapping was applied with the coefficients estimated with 5000 bootstraps.

## 3. Results

### 3.1. Empathy, Positive/Negative Affect, and Personality in Relation to Engagement in Nursing: Descriptive and Correlational Analyses

The correlation coefficients found (Table 1) show positive relationships between cognitive empathy and agreeableness (*r* = 0.26; *p* < 0.001), conscientiousness (*r* = 0.23; *p* < 0.001), and openness to experience (*r* = 0.21; *p* < 0.001). Affective empathy had positive correlation with neuroticism (*r* = 0.20; *p* < 0.001).

In addition, positive affect was positively related to conscientiousness (*r* = 0.20; *p* < 0.001) and neuroticism (*r* = 0.25; *p* < 0.001).

Finally, the associations of personality variables with each of the components of engagement observed were as follows:

The Vigor factor correlated positively with cognitive (*r* = 0.33; *p* < 0.001) empathy, positive affect (*r* = 0.37; *p* < 0.001), extraversion (*r* = 0.20; *p* < 0.001), agreeableness (*r* = 0.27; *p* < 0.001), conscientiousness (*r* = 0.36; *p* < 0.001), and openness to experience (*r* = 0.23; *p* < 0.001).

The Dedication factor was positively correlated with cognitive empathy (*r* = 0.28; *p* < 0.001), positive affect (*r* = 0.36; *p* < 0.001), affability (*r* = 0.24; *p* < 0.001), conscientiousness (*r =* 0.32; *p* < 0.001), and openness to experience (*r* = 0.22; *p* < 0.001).

Absorption was positively related to cognitive empathy (*r* = 0.25; *p* < 0.001) and also positive affect (*r* = 0.35; *p* < 0.001). It was also associated positively with the conscientiousness personality factor (*r =* 0.23; *p* < 0.001).

**Figure 1:**
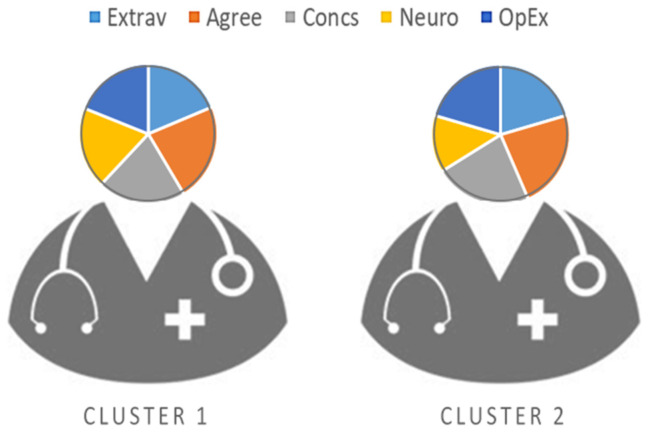
Personality factor profiles.

Based on the data found in the correlational analyses, associations were detected that suggested profiles based on the scores for the personality factors. Therefore, a two-step cluster analysis was performed to identify the personality profiles of the professionals. Two profiles resulted from the inclusion of these variables (Figure 1) with the following distribution: 37.2% (*n* = 472) of the subjects were in Cluster 1, and 62.8% (*n* = 796) were in Cluster 2.

Cluster 1 was characterized by mean scores below those for the total sample in conscientiousness (*M* = 3.34), agreeableness (*M* = 3.67), openness to experience (*M* = 3.07), and extraversion (*M* = 3.03) and above the sample mean in neuroticism (*M* = 3.07). Whereas, in Cluster 2, mean scores were higher than those for the complete sample of professionals in all the personality factors except neuroticism, where the score was below the mean (*M* = 2.44).

After the groups had been classified by the two-cluster solution, a Student’s *t* test for independent samples was done to find out whether there were differences between the clusters with respect to each of the study variables. There were significant differences between the clusters for cognitive empathy (*t*_(1266)_ = −7.76; *p* < 0.001; *d* = 0.45) and positive (*t*_(1266)_ = −6.02; *p* < 0.001; *d* = 0.35) and negative (*t*_(1266)_ = 6.52; *p* < 0.001; *d* = 0.38) affect. No differences were observed between clusters for affective empathy (*t*_(1266)_= 0.98; *p* = 0.326).

In the engagement dimensions, the professionals in Cluster 2 (Vigor: *M* = 4.73, *SD* = 0.92; Dedication: *M* = 4.74, *SD* = 1.06; Absorption: *M* = 4.17, *SD* = 1.04) had significantly higher scores (Vigor: *t*_(1266)_ = −12.12; *p* < 0.001; *d* = 0.71; Dedication: *t*_(1266)_ = −11.27; *p* < 0.001; *d* = 0.66; Absorption: *t*_(1266)_ = −7.17; *p* < 0.001; *d* = 0.42) than Cluster 1 (Vigor: *M* = 3.99, *SD* = 1.11; Dedication: *M* = 3.98, *SD* = 1.20; Absorption: *M* = 3.71, *SD* = 1.11).

### 3.2. Components of Empathy, Positive, and Negative Affect as Predictors of Engagement in Nursing Professionals

As shown in Table 2, the regression analysis provided three models for the Vigor dimension of engagement, of which the last provided the most explained variance with 26.8% (*R*^2^ = 0.26) of the variance explained by variables included in the model. To confirm model validity, residual independence was analyzed. The Durbin–Watson test found *D* = 1.85, confirming an absence of positive and negative correlation. Furthermore, the *t* was found to be associated with a probability of error below 0.05 in all of the variables included in the model. Meanwhile, the standardized coefficients revealed that the variable with the most explanatory weight was positive affect.

Three models were found by the regression analysis for Dedication, where the explained variance of the last was 24.5% (*R*^2^ = 0.24). In this case, the *D* = 1.76 confirmed model validity. The *t* detected association with a probability of error below 0.05 for all the variables included in the model. According to the standardized coefficients found, positive affect was the strongest predictor of Dedication in the study sample. Finally, with regard to Absorption, the regression analysis found four models, where the fourth provided 18.3% of the explained variance (*R*^2^ = 0.18) and the *D* = 1.84 confirmed model validity. The *t* detected an association between the variables with a probability of error below 0.05 for all the variables included. Again, positive affect was the strongest predictor of Absorption. According to the tolerance and variance inflation factor (VIF), an absence of collinearity may be assumed for all the variables in the model.

### 3.3. Mediation Mmodels for Positive Affect and Cognitive Empathy on the Relationship between Personality and the Components of Engagement

In the first place, a statistically significant effect [B = 0.15, *p* < 0.001] of personality (X) on positive affect (M_1_) was observed. The second regression analysis took the result of Mediator 2 (cognitive empathy) as the result variable and included personality (X) and positive affect (M_1_) in the equation. This last has a significant effect [B = 0.46, *p* < 0.01] on cognitive empathy (M_2_) and also personality [B = 1.12, *p* < 0.001]. Table 3 shows the direct, indirect, and total effects, resulting from computation of the mediation models for each of the components of engagement.

**Figure 2:**
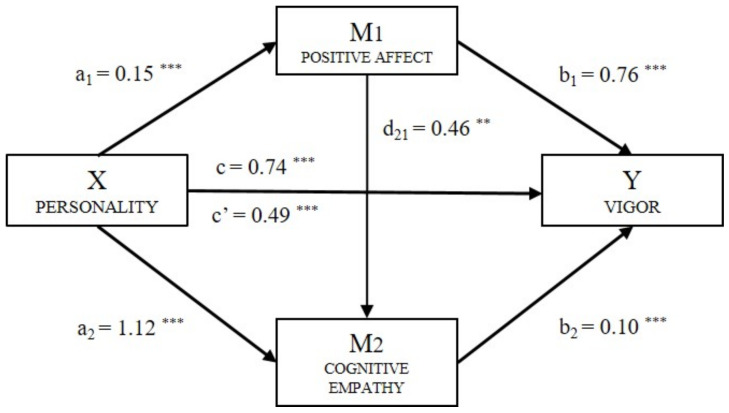
Multiple mediation model of positive affect and cognitive empathy on the relationship between personality and Vigor (Note. ** *p* < 0.01, *** *p* < 0.001).

The effect of the independent variable and the two mediators was estimated with the third regression analysis, taking Vigor (Y_V_) as the result variable (Figure 2). In all cases, significant effects were observed: personality [B = 0.49, *p* < 0.001], positive affect [B = 0.76, *p* < 0.001], and cognitive empathy [B = 0.10, *p* < 0.001]. The direct effect of personality on Vigor was significant [B = 0.49, *p* < 0.001], where the total effect of Model B = 0.74, *p* < 0.001. in the analysis of the indirect effects using bootstrapping, data found for vigor supported a significant level for Path 1 (ind_1_: X → M_1_ → Y_V_; B = 0.118, SE = 0.024, 95% CI (0.073, 0.168)) Path 2 (ind_2_: X → M_1_ → Y_V_; B = 0.007, SE = 0.003, 95% CI (0.000, 0.015))and Path 3 (ind_3_: X → M_1_ → Y_V_; B = 0.116, SE = 0.026, 95% CI (0.072, 0.175)).

**Figure 3:**
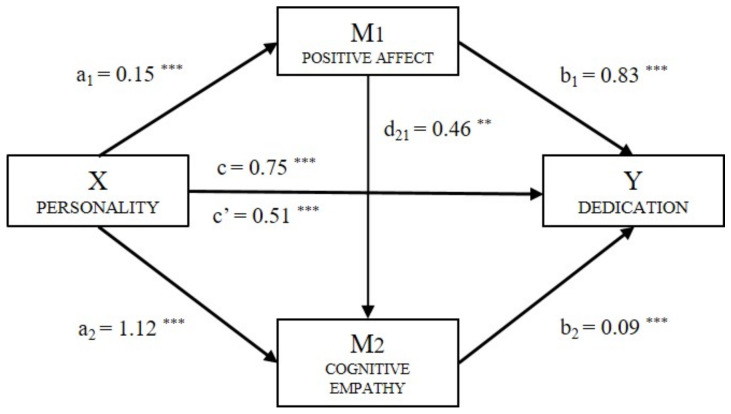
Multiple mediation model of positive affect and cognitive empathy on the relationship between personality and Dedication (Note. ** *p* < 0.01, *** *p* < 0.001).

The Dedication factor (Y_D_) (Figure 3) had a significant effect on personality (B = 0.51, *p* < 0.001), positive affect (B = 0.83, *p* < 0.001), and cognitive empathy (B = 0.09, *p* < 0.001). The total effect of the model (B = 0.75, *p* < 0.001) was also significant. The analysis of the indirect effects of Dedication showed a significant level for Path 1 (ind_1_: X → M_1_ → Y; B = 0.128, SE = 0.026, 95% CI (0.080, 0.184)), Path 2 (ind_2_: X → M_1_ → Y; B = 0.006, SE = 0.003, 95% CI (0.000, 0.014)) and Path 3 (ind_3_: X → M_1_ → Y; B = 0.102, SE = 0.025, 95% CI (0.062, 0.165)).

**Figure 4:**
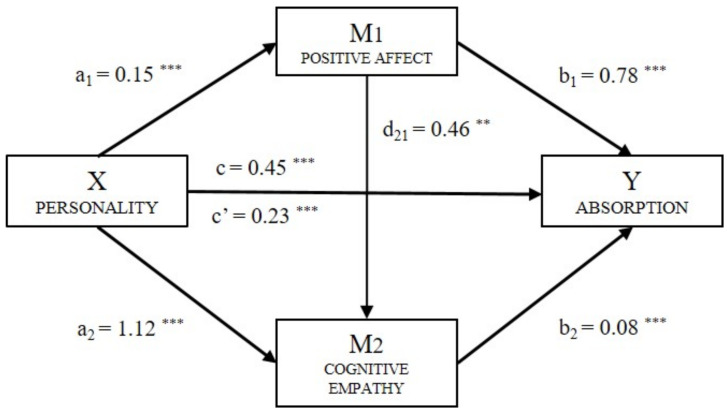
Multiple mediation model of positive affect and cognitive empathy on the relationship between personality and Absorption (Note. ** *p* < 0.01, *** *p* < 0.001).

Finally, for the Absorption factor (Y_A_) (Figure 4), significant effects of personality (B = 0.23, *p* < 0.001), positive affect (B = 0.78, *p* < 0.001), and cognitive empathy (B = 0.08, *p* < 0.001) were observed. The total effect of the model was significant (B = 0.45, *p* < 0.001). Based on the analysis of indirect effects, data were found for Absorption, which support a significant level for Path 1 (ind_1_: X → M_1_ → Y_A_; B = 0.122, SE = 0.024, 95% CI (0.078, 0.172)), Path 2 (ind_2_: X → M_1_ → Y_A_; B = 0.005, SE = 0.003, 95% CI (0.000, 0.013)) and Path 3 (ind_3_: X → M_1_ → Y_A_; B = 0.093, SE = 0.023, 95% CI (0.056, 0.149)).

## 4. Discussion

The first objective posed in this study was to analyze the relationships between the components of empathy, affect, personality, and engagement in nursing professionals. As shown in the results, the engagement components showed a positive relationship with empathy (both cognitive and affective) and positive affect. A positive relationship was observed in the Big Five factors with agreeableness, and there was a tendency toward the same sign with the rest of the factors except neuroticism, which was negative. These results are in agreement with what has been found by other authors who mention that the Big Five personality factors show a positive relationship with wellbeing and work engagement [21,22], except for neuroticism, which has the opposite relationship [14].

Concerning the association found between the engagement factors and empathy, the positive relationship may be explained by empathy having been shown to be especially relevant in preventing burnout and distress in nursing personnel [32,33]. However, the correlation analysis showed a positive relationship between the vigor factor and absorption in engagement and both cognitive and affective empathy, whereas dedication showed only a positive relationship with the cognitive component of empathy. The results are partially supported by the study by Macysiak et al. [30], who mentioned that while cognitive empathy improved satisfaction and work engagement, the affective dimension did not.

Furthermore, in regard to the second objective posed, the cluster analysis showed the existence of two groups of nursing professionals with different personality profiles. The first group was made up of individuals with levels below the mean in all the Big Five personality factors, except neuroticism, where the values were above the mean. On the contrary, the second group was comprised of professionals who had scores above the mean in extraversion, agreeableness, openness to experience, and conscientiousness, and below it in neuroticism. This second group, which was also more numerous, had significantly higher scores in positive and negative affect, cognitive empathy, and engagement. These results support the findings of an earlier study in which nursing professionals who showed low scores in neuroticism and high in the other Big Five factors had more work engagement [34]. These personality profiles found in nursing professionals, specifically the one framed by high scores in all the factors except neuroticism, could be related to a resilient personality, which improves coping with the stressful situations typical in this sector [20]. The fact that it was in this group of professionals where higher cognitive empathy was found could also be related to modulation of the influence of stress in these professionals by the cognitive factor of empathy [31].

Third, the variables with the most explanatory value for the engagement factors were analyzed. The results showed that for both vigor and dedication, the model that best explained the scores on these factors was made up of the cognitive empathy, negative affect, and positive affect variables. Specifically, the last component showed the highest predictive value for the two engagement factors. Similarly, with respect to absorption, the model that best explained the scores on this factor was made up of the same variables as for the dedication and vigor factors (that is, cognitive empathy, positive and negative affect), and also affective empathy. Again, in this case, positive affect was the component that best explained absorption.

The factorial composition of the models proposed is in agreement with what was proposed by Altuwairqi et al. [42], who found that positive affective states act as predictors of the levels of occupational engagement. However, that model was analyzed in a sample of students in the area of technology, so in future studies, it would be suitable to go deeper into this affective model of engagement and its application in the healthcare context. The especially important role of positive affect on the components of nursing engagement may be due to this variable promoting the presence of positive emotions and also in the work environment. Thus, it is the characteristics of the work itself performed in the profession, such as continuous social interaction (with patients, family members, coworkers, supervisors, and so on), that could be related to the results found for positive affect. We think that often feeling optimistic and positive at work helps to be engaged with it and motivated to face the challenges that could arise.

Finally, due to the need to explain work engagement in nursing by understanding the variables involved and their relationships [53], mediation models for personality were analyzed on each of the engagement factors. The results showed that personality influenced engagement positively, and this effect was mediated mainly by cognitive empathy. Thus, we found that personality affected the vigor, dedication, and absorption factors of engagement, and that cognitive empathy mediated this relationship. This follows in the line of the results of other studies, where cognitive empathy exerted a strong influence on strategies and emotion control, which is necessary to increase the commitment of nursing professionals [14,43,47]. Eley et al. [66] noted that the main reason for nurses taking up the profession is helping others, and they found the motivation between choice of profession, personality traits, and empathy to be congruent. Therefore, certain personality traits and empathy would be dominant characteristics in professionals who feel passion and engagement with their nursing work. However, in this sense, as Harris [67] noted, the effects of empathy’s cognitive and affective domains are different in different people’s behavior. Thus, our results suggest that cognitive empathy, or in other words, the ability to recognize and understand emotions, mediates more strongly between personality and engagement than emotional empathy (that is, the emotional reaction to social stimuli). Probably because in the care environment, understanding the patient promotes an engaged attitude with the work of caring, which is more important than showing emotional empathy, since the emotional factor of empathy can affect engagement with some patients with whom less closeness is created and cause stress in nurses when negative feelings or anguish appear [68].

It was also found that positive affect mediated between personality and the engagement factors, although it was weaker. This is in agreement with the proposal of Barr [50], who found that personality affects the level of commitment of nursing personnel, which is mediated by mood and affective states. According to Fredrickson [69], discrete positive emotions in the workplace facilitate the construction of psychological resources that promote effective functioning and engagement with one’s work. Therefore, in the future, classification of specific positive emotions that help improve attitudes and work results, beyond the division between positive and negative affect, should be studied [70].

## 5. Conclusions

In recent times, we have seen social concern related to job turnover and quitting, especially accentuated in the healthcare sector. Due to the effects of engagement on this problem, as well as the many benefits, to professionals, patients, and to the organization, it becomes indispensable to analyze the variables related to its levels in nursing workers. This study showed that empathy, affect, and personality influence the factors of engagement in nursing professionals.

Among the limitations of this study, it should be mentioned that sociocultural variables were not included. Neither did it take into account the department where the professionals were working, which could be related to the feelings of empathy they develop toward patients and family members cared for, as well as affective states experienced depending on the different situations they are faced with. Therefore, it would be advisable to take this variable into consideration in future studies. In addition, data were collected by online questionnaires, which may have generated participation of the participants for reasons of interest and would have repercussions, at least partly, on the results found.

This study shows the need to continue investigating the factors that affect and mediate in engagement of nursing professionals. Since empathy and affect, which are potentially modifiable, have shown their influence on the performance of these workers, their intervention could imply increased engagement and wellbeing of healthcare professionals, with repercussions in turn on patients and the organization as a whole. The implications for nursing practice are materialized in the contributions that this type of study can offer for the design of effective intervention to improve the socioemotional sphere, which in this profession is of special importance. Business leaders should strengthen socioemotional skills already present in most cases for the ultimate goal of achieving the personal well-being of the professionals, by promoting work environments optimum for their development.

## Figures and Tables

**Table 1:** Descriptive and correlational statistics: empathy, affect, personality, and engagement (*n* = 1268).

	M	SD	1	2	3	4	5	6	7	8	9	10	11
1. Cognitive empathy	19.36	2.55	–										
2. Affective	13.36	3.02	0.33 ***	–									
3. Positive affect	2.99	0.43	0.11 ***	0.03	–								
4. Negative affect	2.09	0.39	−0.06 *	0.09 **	−0.47 ***	–							
5. Extraversion	3.50	0.80	0.13 ***	−0.02	0.12 ***	−0.07 *	–						
6. Agreeableness	4.01	0.58	0.260 ***	0.09 **	0.10 ***	−0.10 ***	0.21 ***	–					
7. Conscientiousness	3.87	0.67	0.23 ***	−0.01	0.20 ***	−0.14 ***	0.17 ***	0.23 ***	–				
8. Neuroticism	2.67	0.81	−0.09 **	0.20 ***	−0.14 ***	0.25 ***	−0.19 ***	−0.14 ***	−0.25 ***	–			
9. Openness to experience	3.51	0.74	0.21 ***	0.00	0.17 ***	−0.06 *	0.13 ***	0.13 ***	0.29 ***	−0.14 ***	–		
10. Vigor	4.46	1.06	0.33 ***	0.07 **	0.37 ***	−0.02	0.20 ***	0.27 ***	0.36 ***	−0.18 ***	0.23 ***	–	
11. Dedication	4.45	1.17	0.28 ^***^	0.04	0.36 ***	−0.05 *	0.19 ***	0.24 ***	0.32 ***	−0.19 ***	0.22 ***	0.90 ***	–
12. Absorption	4.00	1.09	0.25 ***	0.12 ***	0.35 ***	0.05 *	0.11 ***	0.19 ***	0.23 ***	−0.06 *	0.16 ***	0.83 ***	0.82 ***

* *p* < 0.05; ** *p* < 0.01; *** *p* < 0.001.

**Table 2:** Stepwise multiple linear regression model (*N* = 1268).

**VIGOR**	**Model**	***R***	***R*^2^**	**Corrected *R*^2^**			**Change Statistics**											**Durbin Watson**
							**Standard Error of Estimation**			**Change in *R*^2^**	**Change in *F***			**Sig. of Change in *F***				
	1	0.377	0.142	0.142			0.98			0.14	208.82			0.000				1.85
	2	0.478	0.228	0.227			0.93			0.08	139.93			0.000				
	3	0.518	0.268	0.267			0.91			0.04	68.97			0.000				
	Model 4		Unstandardized coefficients						Standardized coefficients		*t*	Sig.					Collinearity	
			*B*		Std. error				Beta								Tol.	VIF
	(Constant)		0.21		0.26						0.81	0.418						
	Positive affect		1.13		0.06				0.45		16.39	0.000					00.74	1.33
	Cognitive empathy		0.11		0.01				0.26		10.87	0.000					0.96	1.03
	Negative affect		−0.61		0.07				−0.23		−8.30	0.000					0.75	1.32
**DEDICATION**	**Model**	***R***	***R*^2^**	**Corrected *R*^2^**				**Change Statistics**										**Durbin Watson**
								**Standard Error of Estimation**		**Change in *R^2^***	**Change in *F***			**Sig. of Change in *F***				
	1	0.365	0.133	0.132				1.09		0.13	193.03			0.000				1.76
	2	0.450	0.203	0.201				1.05		0.07	109.57			0.000				
	3	0.495	0.245	0.243				1.02		0.04	71.00			0.000				
	Model 4		Unstandardized coefficients						Standardized coefficients		*t*		Sig.			Collinearity		
			*B*			Std. error			Beta							Tol.		VIF
	(Constant)		0.40			0.29					1.35		0.176					
	Positive affect		1.28			0.07			0.46		16.54		0.000			0.74		1.33
	Negative affect		−0.79			0.08			−0.26		−9.47		0.000			0.75		1.32
	Cognitive empathy		0.09			0.01			0.21		8.42		0.000			0.96		1.03
**ABSORPTION**	**Model**	***R***	***R*^2^**	**Corrected *R*^2^**				**Change Statistics**										**Durbin Watson**
								**Standard Error of Estimation**		**Change in *R^2^***	**Change in *F***			**Sig. of Change in *F***				
	1	0.352	0.124	0.123				1.02		0.12	177.90			0.000				1.84
	2	0.413	0.170	0.169				0.99		0.04	70.13			0.000				
	3	0.424	0.180	0.178				0.98		0.01	14.63			0.000				
	4	0.427	0.183	0.180				0.98		0.00	4.50			0.034				
	Model 4		Unstandardized coefficients						Standardized coefficients		*t*	Sig.			Collinearity			
			*B*			Std. error			Beta						Tol.			VIF
	(Constant)		−0.02			0.28					−0.07	0.937						
	Positive affect		0.98			0.07			0.38		13.08	0.000			0.74			1.34
	Cognitive empathy		0.07			0.01			0.18		6.59	0.000			0.85			1.17
	Negative affect		−0.33			0.08			−0.12		−4.08	0.000			0.74			1.35
	Affective empathy		0.02			0.01			0.05		2.12	0.034			0.87			1.14

**Table 3:** Direct, total, and indirect effects: Vigor, Dedication, and Absorption.

Vigor (VI)	B	SE	t	95% CI
Direct Effect Personality → VI	0.499 ***	0.055	9.085	(0.391, 0.607)
Total Effect Personality → VI	0.741 ***	0.058	12.671	(0.626, 0.856)
Ind 1: Personality → PA → VI	0.118	0.024		(0.073, 0.168)
Ind 2: Personality → PA → CE → VI	0.007	0.003		(0.000, 0.015)
Ind 3: Personality → CE → VI	0.116	0.026		(0.072, 0.175)
**Dedication (DE)**	**B**	**SE**	**t**	**95% CI**
Direct Effect Personality → DE	0.519 ***	0.062	8.313	(0.397, 0.642)
Total Effect Personality → DE	0.758 ***	0.065	11.612	(0.630, 0.886)
Ind 1: Personality → PA → DE	0.128	0.026		(0.080, 0.184)
Ind 2: Personality → PA → CE → DE	0.006	0.003		(0.000, 0.014)
Ind 3: Personality → CE → DE	0.102	0.025		(0.062, 0.165)
**Absorption (AB)**	**B**	**SE**	**t**	**95% CI**
Direct Effect Personality → AB	0.230 ***	0.059	3.843	(0.112, 0.347)
Total Effect Personality → AB	0.451 ***	0.062	7.253	(0.329, 0.573)
Ind 1: Personality → PA → AB	0.122	0.024		(0.078, 0.172)
Ind 2: Personality → PA → CE → AB	0.005	0.003		(0.000, 0.013)
Ind 3: Personality → CE → AB	0.093	0.023		(0.056, 0.149)

Note: Ind: indirect effect, SE = standard error, CI = confidence interval, B = non-standardized regression coefficient; Only confidence intervals were considered for the indirect effects. *** *p* < 0.001.

## Data Availability

The data that support the findings of this study are available from the corresponding author upon reasonable request.

## References

[1] De Simone S., Planta A., Cicotto G. (2018). The role of job satisfaction, work engagement, self-efficacy and agentic capacities on nurses’ turnover intention and patient satisfaction. Appl. Nurs. Res..

[2] Eltaybani S., Noguchi-Watanabe M., Igarashi A., Saito Y., Yamamoto-Mitani N. (2018). Factors related to intention to stay in the current workplace among long-term care nurses: A nationwide survey. Int. J. Nurs. Stud..

[3] Orgambídez-Ramos A., de Almeida H. (2017). Work engagement, social support, and job satisfaction in Portuguese nursing staff: A winning combination. Appl. Nurs. Res..

[4] Lisbona A., Palaci F., Salanova M., Frese M. (2018). The effects of work engagement and self-efficacy on personal initiative and performance. Psicothema.

[5] Patrick H.A., Mukherjee U. (2018). Work engagement: A cross sectional study of employees in the healthcare sector. Contemp. Manag. Res..

[6] Han S.S., Han J.W., Kim Y.H. (2018). Effect of Nurses’ Emotional Labor on Customer Orientation and Service Delivery: The Mediating Effects of Work Engagement and Burnout. Saf. Health Work.

[7] Schaufeli W.B. (2015). Engaging leadership in the job demands-resources model. Career Dev. Int..

[8] Schaufeli W.B., Taris T.W., Bauer G.F., Hämmig O. (2014). A critical review of the job demands-resources model: Implications for improving work and health. Bridging Occupational, Organizational and Public Health.

[9] García-Sierra R., Fernández-Castro J., Martínez-Zaragoza F. (2017). Engagement of nurses in their profession. Qualitative study on engagement. Enferm. Clin..

[10] Sonnentag S. (2017). A task-level perspective on work engagement: A new approach that helps to differentiate the concepts of engagement and burnout. Burn. Res..

[11] Pérez-Fuentes M.C., Molero M.M., Gázquez J.J., Oropesa N.F. (2018). The Role of Emotional Intelligence in Engagement in Nurses. Int. J. Environ. Res. Public Health.

[12] Chaudhary R., Akhouri A. (2018). Linking corporate social responsibility attributions and creativity: Modeling work engagement as a mediator. J. Clean. Prod..

[13] Tirado G., Llorente-Alonso M., Topa G. (2019). Desequilibrio esfuerzo-recompensa y quejas subjetivas de salud: Estudio exploratorio entre médicos en España [Effort-reward imbalance and subjective health complaints: An exploratory study among doctors in Spain]. Eur. J. Investig. Health Psychol. Educ..

[14] Van Mol M.M.C., Nijkamp M.D., Bakker J., Schaufeli W.B., Kompanje J.O. (2018). Counterbalancing work-related stress? Work engagement among intensive care professionals. Aust. Crit. Care.

[15] Lorraine K., Cianelli R. (2014). Exploring the concept of nurse engagement related to patient experience. Horiz. Enferm..

[16] Martos Á., Pérez-Fuentes M.C., Gázquez J.J., Simón M.M., Barragán A.B. (2018). Burnout y engagement en estudiantes de Ciencias de la Salud [Burnout and engagement in students of health sciences]. Eur. J. Investig. Health Psychol. Educ..

[17] Chinelato R.S., de Oliveira S.M., Ferreira M.C., Valentini F. (2020). Perception of organizational politics, psychological safety climate, and work engagement: A cross-level analysis using hierarchical linear modeling. An. Psicol..

[18] Dewe P., Cary L.C. (2017). Work, Stress and Coping.

[19] Goldberg L. (1993). The structure of phenotypic personality traits. Am. Psychol..

[20] Törnroos M., Hintsanen M., Hintsa T., Jokela M., Pulkki-Råback L., Hutri-Kähönen N., Keltikangas-Järvinen L. (2013). Associations between Five-Factor Model traits and perceived job strain: A population-based study. J. Occup. Health Psychol..

[21] Bagley C., Abubaker M., Sawyer A. (2018). Personality, Work-Life Balance, Hardiness, and Vocation: A Typology of Nurses and Nursing Values in a Special Sample of English Hospital Nurses. Adm. Sci..

[22] Pérez-Fuentes M.C., Molero M.M., Martos Á., Gázquez J.J. (2018). Burnout and Engagement: Personality Profiles in Nursing Professionals. J. Clin. Med..

[23] McKinnon J. (2018). In their shoes: An ontological perspective on empathy in nursing practice. J. Clin. Nurs..

[24] Bourgault P., Lavoie S., Paul-Savoie E., Grégoire M., Michaud C., Gosselin E., Johnston C. (2015). Relationship Between Empathy and Well-Being Among Emergency Nurses. J. Emerg. Nurs..

[25] Pérez-Fuentes M.C., Molero M.M., Gázquez J.J. (2019). Explanatory Value of General Self-Efficacy, Empathy and Emotional Intelligence in Overall Self-Esteem of Healthcare Professionals. Soc. Work Public Health.

[26] Hojat M., Louis D.Z., Maio V., Gonnella J.S. (2013). Empathy and health care quality. Am. J. Med. Qual..

[27] Oh J. (2019). Effects of Nursing Students’ Empathy and Interpersonal Competence on Ideal Nurse Attributes. J. Nurs. Educ..

[28] Levett-Jones T., Cant R., Lapkin S. (2019). A systematic review of the effectiveness of empathy education for undergraduate nursing students. Nurse Educ. Today.

[29] Teding van Berkhout T., Malouff J.M. (2016). The efficacy of empathy training: A meta-analysis of randomized controlled trials. J. Couns. Psychol..

[30] Macysiak M., Dabrowska O., Marcysiak M.B. (2014). Understanding the concept of empathy in relation to nursing. Prog. Health Sci..

[31] Vioulac C., Aubree C., Massy Z., Untas A. (2016). Empathy and stress in nurses working in haemodialysis: A qualitative study. J. Adv. Nurs..

[32] Ferri P., Guerra E., Luigi M., Cunico L., Di Lorenzo R. (2015). Empathy and burnout: An analytic cross-sectional study among nurses and nursing students. Acta Biomed..

[33] Salvarani V., Rampoldi G., Ardenghi S., Bani M., Blasi P., Ausili D., Di Mauro S., Strepparava M.G. (2019). Protecting emergency room nurses from burnout: The role of dispositional mindfulness, emotion regulation and empathy. J. Nurs. Manag..

[34] Pérez-Fuentes M.C., Molero M.M., Gázquez J.J., Simón M.M. (2019). Analysis of Burnout Predictors in Nursing: Risk and Protective Psychological Factors. Eur. J. Psychol. Appl. Leg. Context.

[35] Stephens M., Ormandy P. (2019). An Evidence-based Approach to Measuring Affective Domain Development. J. Prof. Nurs..

[36] Martínez-Iñigo D., Bermejo-Pablos C., Totterdell P. (2018). The boomerang effect: How nurses’ regulation of patients’ affect associates with their own emotional exhaustion and affective experiences. Int. J. Stress Manag..

[37] Watson D., Clark L.A., Tellegen A. (1988). Development and validation of brief measures of Positive and Negative Affect: The PANAS scales. J. Pers. Soc. Psychol..

[38] Hernández G.P., Rovira T., Quirin M., Edo S. (2020). A Spanish adaptation of the implicit positive and negative affect test (IPANAT). Psicothema.

[39] Ros-Morente A., Alsinet C., Torrelles C., Blasco-Belled A., Jordana N. (2018). An examination of the relationship between Emotional Intelligence, Positive Affect Character Strengths and Virtues. An. Psicol..

[40] Teismann T., Brailovskaia J., Margraf J. (2019). Positive mental health, positive affect and suicide ideation. Int. J. Clin. Health Psychol..

[41] Van Bogaert P., Peremans L., Van Heusden D., Verspuy M., Kureckova V., Van de Cruys Z., Franck E. (2017). Predictors of burnout, work engagement and nurse reported job outcomes and quality of care: A mixed method study. BMC Nurs..

[42] Altuwairqi K., Jarraya S.K., Allinjawi A., Hammami M. (2021). A new emotion–based affective model to detect student’s engagement. J. King Saud. Univ. Comp. Inf. Sci..

[43] Wu X., Li J., Liu G., Liu Y., Cao J., Juan Z. (2018). The effects of emotional labor and competency on job satisfaction in nurses of China: A nationwide cross-sectional survey. Int. J. Nurs. Sci..

[44] Morales F. (2020). Coping strategies, empathy and prosocial tendencies in university students. Eur. J. Psychol. Educ..

[45] Mestre V., Frías M.D., Samper P. (2004). Measuring empathy: The Interpersonal Reactivity Index. Psicothema.

[46] Navarro-Abal Y., López-López M.J., Climent-Rodríguez J.A. (2018). Engagement, resilience and empathy in nursing assistants. Enferm. Clin..

[47] Cao X., Chen L. (2019). Relationships among social support, empathy, resilience and work engagement in haemodialysis nurses. Int. Nurs. Rev..

[48] Wang C., Wu Q., Feng M., Wan Q., Wu X. (2017). International Nursing: Research on the Correlation between Empathy and China’s Big Five Personality Theory: Implications for Nursing Leaders. Nurs. Adm. Q..

[49] Muglia S., Benson N., Machado W.L., Bachert C.M.A., Fernandes E. (2018). Adult temperament styles: A network analysis of their relationships with the Big Five Personality Model. Eur. J. Educ. Psychol..

[50] Barr P. (2018). Personality Traits, State Positive and Negative Affect, and Professional Quality of Life in Neonatal Nurses. J. Obstet. Gynecol. Neonatal Nurs..

[51] Wang Z., Li C., Li X. (2017). Resilience, Leadership and Work Engagement: The Mediating Role of Positive Affect. Soc. Indic. Res..

[52] Cao Y., Liu J., Yang M., Liu Y. (2019). The mediating role of organizational commitment between calling and work engagement of nurses: A cross-sectional study. Int. J. Nurs. Sci..

[53] Lu H., Zhao Y., While A. (2019). Job satisfaction among hospital nurses: A literature review. Int. J. Nurs. Stud..

[54] Schaufeli W.B., Bakker A.B. (2003). UWES Utrecht Work Engagement Scale Preliminary Manual.

[55] Schaufeli W.B., Salanova M., González-Romá V., Bakker A.B. (2002). The measurement of engagement and burnout: A two sample confirmatory factor analytic approach. J. Happiness Stud..

[56] Rammstedt B., John O.P. (2007). Measuring personality in one minute or less: A 10-item short version of the Big Five Inventory in English and German. J. Res. Pers..

[57] John O.P., Donahue E.M., Kentle R.L. (1991). The Big Five Inventory-Versions 4a and 54.

[58] John O.P., Srivastava S., Pervin L.A., John O.P. (1999). The Big Five trait taxonomy: History, measurement, and theoretical perspectives. Handbook of Personality Theory and Research.

[59] Rammstedt B., Kemper C.J., Klein M.C., Beierlein C., Kovaleva A. (2013). A Short Scale for Assessing the Big Five Dimensions of Personality-10 Item Big Five Inventory (BFI-10). Methods Data Anal..

[60] Rammstedt B., Kemper C.J., Klein M.C., Beierlein C., Kovaleva A. (2014). Big Five Inventory (BFI-10). Zusammenstellung Sozialwissenschaftlicher Items Und Skalen.

[61] Jolliffe D., Farrington D.P. (2006). Development and validation of the Basic Empathy Scale. J. Adolesc..

[62] Oliva A., Antolín L., Pertegal M., Ríos M., Parra A., Hernando A., Reina M. (2011). Instrumentos para la Evaluación de la Salud Mental y el Desarrollo Positivo Adolescente y los Activos que lo Promueven [Instruments for the Assessment of Adolescent Mental Health and Positive Development and the Assets that Promote it].

[63] Joiner T., Sandin B., Chorot P., Lostao L., Marquina G. (1997). Development and Factor Analytic Validation of the SPANAS among Women in Spain: (More) Cross-Cultural Convergence in the Structure of Mood. J. Pers. Assess..

[64] Cohen J. (1988). Statistical Power Analysis for the Behavioral Sciences.

[65] Hayes A.F. (2013). Introduction to Mediation, Moderation, and Conditional Process. Analysis: A Regression-Based Approach.

[66] Eley D., Eley R., Bertello M., Rogers-Clark C. (2012). Why did I become a nurse? Personality traits and reasons for entering nursing. Int. J. Nurs. Pract..

[67] Davis M.H. (1983). A Multidimensional Approach to Individual Differences in Empathy. J. Pers. Soc. Psychol..

[68] Yegdich T. (2001). On the phenomenology of empathy in nursing: Empathy or sympathy?. J. Adv. Nurs..

[69] Fredrickson B.L. (2001). The role of positive emotions in positive psychology: The broaden-and-build theory of positive emotions. Am. Psychol..

[70] Camiseta Y.J. (2019). Uncovering the trail of positive affect in the job attitudes literature: A systematic review. Asian J. Soc. Psychol..

